# The Role of miRNA-221 and miRNA-34a in Non-Melanoma Skin Cancer of the Head and Neck Region

**DOI:** 10.3390/genes14020503

**Published:** 2023-02-16

**Authors:** Tiberiu Tamas, Lajos Raduly, Ioana Berindan-Neagoe, Cristian Dinu, Emil Botan, Bogdan Bumbu, Adela Tamas, Sebastian Stoia, Daniel Corneliu Leucuta, Simion Bran, Florin Onisor, Grigore Băciuț, Gabriel Armencea, Mihaela Băciuț

**Affiliations:** 1 Department of Maxillofacial Surgery and Implantology, Faculty of Dentistry, “Iuliu Hațieganu” University of Medicine and Pharmacy, 400012 Cluj-Napoca, Romania; 2 Research Center for Functional Genomics, Biomedicine and Translational Medicine, “Iuliu Hațieganu” University of Medicine and Pharmacy, 400012 Cluj-Napoca, Romania; 3 Department of Pathology, Emergency County Hospital, 400347 Cluj-Napoca, Romania; 4 Department of Oral Surgery, Dental Medicine, Faculty of Medicine and Pharmacy, University of Oradea, 410087 Oradea, Romania; 5 Doctoral School, “Iuliu Hatieganu” University of Medicine and Pharmacy, 400012 Cluj-Napoca, Romania; 6 Medical Informatics and Biostatistics Department, “Iuliu Hatieganu” University of Medicine and Pharmacy, 400012 Cluj-Napoca, Romania

**Keywords:** non-melanoma skin cancer, head and neck, BCC, SCC, BSC, miRNA, non-coding RNA

## Abstract

Non-melanoma skin cancer (NMSC) is one of the most frequent types of malignancy in the human body with an increasing incidence. Short, non-coding RNA molecules called microRNAs (miRNAs) can control post-transcriptional gene expression and they have a significant role in several physiological cellular processes and pathologies, including cancer. Depending on the functions of the genes, miRNAs may function as oncogenes or tumor suppressors. The aim of this paper was to describe the role of miRNA-34a and miRNA-221 in head and neck NMSC. Thirty-eight NMSC match paired (tumor and adjacent) tissue samples were evaluated by qRT-PCR. Total RNA was extracted and isolated from tissue samples using the phenol-chloroform (Trireagent) method according to the manufacturer’s protocol. The concentration of RNA was measured by a NanoDrop-1000 spectrophotometer. The expression level of each miRNA was calculated by threshold cycle. For all statistical tests, the 0.05 significance level was used and two-tailed p values. All analyses were conducted in an R environment for statistical computing and graphics. We found the miRNA-221 being overexpressed in squamous cell carcinoma (SCC) (*p* < 0.05), basal cell carcinoma (BCC) and basosquamous cell carcinoma (BSC) compared with adjacent normal tissue. Additionally, the levels of miRNA-221 were two times higher (*p* < 0.05) in cases where the excision of the tumor was done with positive margins (R1), which means that we are the first to highlight the potential role of miRNA-221 in the microscopical local invasion. Mi-RNA-34a expression was altered in the malignant tissue compared with the adjacent normal one both in BCC and SCC but not statistically significantly. In conclusion, NMSC are challenging because of their increasing incidence and rapidly evolving development and discovering their molecular mechanisms of action lead us to understand tumorigenesis and evolution, while also contributing to the implementation of novel therapeutic keys.

## 1. Introduction

The skin represents the largest organ in the human body and serves as first line of protection against diverse aggression factors. Exposure to the environmental factors, such as ultraviolet (UV) radiation or chemicals, can damage this defensive line, subsequently developing skin cancer [1,2,3]. The incidence is quickly rising globally for both melanoma and non-melanoma cancers (NMSC). In 2020, over 1 million new cases have been identified worldwide, ranking NMSC on the fourth place among all cancers, according to the Globocan statistical data. Compared with melanoma or other types of cancer, the mortality rate is lower [4,5,6,7,8]. There are several subtypes, such as cutaneous squamous cell carcinoma (SCC), basal cell carcinoma (BCC), basosquamous cell carcinoma (BSC), and Merkel cell carcinoma (MCC). Surgical treatment consisting of large excision and defect reconstruction is considered the gold standard of treatment for all these tumors, but novel strategies are developing [9,10,11,12,13].

MicroRNAs (miRNAs) are short, non-coding RNA molecules made up of 17 to 25 nucleotides (nt) which can control post-transcriptional gene expression [14,15]. Being encoded within exons and introns, they have a significant importance not only in numerous physiological cellular functions, but also in the development and evolution of diseases, including malignant tumors. They can control gene expression by connecting with the 3′-untranslated regions (3′-UTRs) of target messenger RNAs (mRNAs), which can regulate transcription [16,17,18]. MiRNAs are engaged in a lot of essential biological activities by controlling the protein expression of their target genes. Depending on the functions of the genes, miRNAs may act as new oncogenes or as tumor suppressors [19,20,21].

MiRNAs have the ability to control both protein translation and gene expression [22,23]. A variety of cancer cells actively secrete them into the bloodstream, therefore MiRNAs can be identified in biofluids, such cerebrospinal fluid, serum, plasma, saliva, and urine, as well as in tissue samples [24,25,26,27]. Due to the accessible method of identification, circulating miRNAs are frequently considered the first option in the clinical context, but the results obtained from the body fluids may not be as accurate as those from tissue samples [28,29,30].

The most common NMSC type is the BCC. Numerous miRNA subtypes were connected to malignancies in the literature, especially with the distinction between nodular and infiltrative BCCs. Some variants of advanced BCC or subtypes of BCC, such as the morpheus type, are associated with a very bad prognosis, even though metastasis is uncommon. In SCC, several miRNAs are up- or down-regulated, contributing to the development of various pathological processes. Regional metastases are more common in SCC than in BCC and might have a poor prognosis, as we already know [2,9,10,11,12,13]. Different types of miRNAs were affected by and linked to UVA or UVB radiation in SCC and were particularly connected to the malignant process in comparison with their expression in healthy skin. By targeting particular genes, such as PTEN (phosphatase and tensin homolog), TP53, VEGFA (vascular endothelial growth factor A), MMP13 (matrix metalloproteinase-13), and LZTS1 (leucine zipper putative tumor suppressor 1), miRNAs can also exhibit their inhibitory or stimulating roles in the malignant landmark, involving a synergistic mechanism [2].

MiRNA-221 is a member of the miRNA-221/222 cluster situated on the X chromosome. The miRNA-221/222 gene cluster location, which is carried as far as 726bp, is located on chromosome Xp11.3 in human DNA (deoxyribonucleic acid). The nucleotide sequences of the genes are split into homologous groups and the duplication of a gene’s ancestor’s DNA is how genes paralogously arise. Two classical TATA boxes are located on the 550 and 190 base pairs (bp) upstream and downstream of pre-miRNA-222, respectively, in the promoter region of the miRNA-221/222 genes, while three polyadenylation signals are located downstream of pre-miRNA-221. Angiotensin II is involved in regulating the expression of the miRNA-221/222 gene cluster. A variety of human malignancies, including hepatocellular carcinoma, prostate cancer, and colon cancer, have an up-regulated expression of miRNA-221, enhancing its role in the growth and spread of the malignant process [31,32]. On the other hand, in other cancer sites, such as lung cancer, miRNA-221 has a suppressing effect. Therefore, exploring the role of miRNA-221 in various cancer types is an essential pathway in tumorigenesis [32,33].

The method of miRNA-221/222 production in the nucleus requires the transcription of a lengthy precursor (pri-miRNA-221/222), which is subsequently processed via a more particular area of Dorsha/DiGeorge gene 8 as a nuclear protein. This process generates the 110-nucleotide precursor (pre-miRNA-221/222). Pre-miRNAs are first processed by an endoribonuclease of the RNase III family before being transported into the cytoplasm with the help of the nuclear transporter exportin-5 as a Ran-GTP protein binding to RNA. The Dicer cleaves miRNA precursors to produce mature miRNA duplexes. As an RNA-induced silencing complex, Argonaute-2 (Ago-2)/TAR RNA-binding protein (TRBP)/protein kinase R-activating protein (PACT) stabilizes each miRNA duplex (RISC). Typically, a mature miRNA strand plus a passenger strand without a stem-loop structure make up a double-stranded miRNA, which is an incomplete duplex of transitory molecules. Dicer cleaves the duplex as the RISC complex (miRISC) joins a functioning mature single-stranded miRNA. Other proteins, such as Metadherin (MTDH), glycine-tryptophan protein of 182 KDa (GW182), and staphylococcal nuclease domain-containing protein 1 (SND1), play a notable part in the formation of the miRISC complex [34,35,36].

There are three main miRNA families which restrict tumor growth, as follows: let-7, miRNA-200, and miRNA-34, derived into MiRNA-34a, miRNA-34b, and miRNA-34c. While miRNA-34b and miRNA-34c are produced from a polycistronic transcript encoded on chromosome 11q23.1, miRNA-34a is encoded in the second exon of a gene found on chromosome 1p36.22. The mature miRNA-34a sequence has a length of 22 nucleotides and expresses a high percentage homology with miRNA-34b and miRNA-34c. These results enlighten the possibility that the targets of the various miRNA-34 family members are similar, and the members of the family may therefore be functionally redundant. In normal human tissues, miRNA-34a is mostly expressed, while miRNA-34b/c are downregulated. The seed sequence for miRNA-34a in both humans and mice is 5′-GGCAGUGU-3′ and in several solid tumors and hematological malignancies, miRNA-34a expression is lost. Research has shown that miRNA-34a is a key modulator of p53 functions and a strong tumor suppressor, since miRNA-34 was discovered to be a p53 target. MiRNA-34a inhibits numerous tumor-promoting processes, such as the cell cycle, epithelial-to-mesenchymal transition (EMT), metastasis, stemness, and tumor immunity, as well as tumor-inhibitory events, including apoptosis and senescence. MiRNA-34a controls these biological functions by suppressing target mRNAs, and so far more than 200 miRNA-34a targets have been identified and/or verified [37].

As described before, miRNA-34a is considered a tumor suppressor and it may control the cell cycle and prevent tumor cells from migrating, spreading, and invading adjacent tissue [38,39]. Many malignant tumors, including breast and esophageal cancer, are tightly correlated with miRNA-34a in their occurrence and evolution. MiRNA-34a inactivation may be a protection strategy employed by the tumor cells [40,41,42]. The low expression level is a predictive risk factor for a variety of cancer sites, including lung adenocarcinoma and esophageal tumors. However, the role of miRNA-34a in NMSC is not well defined [42,43].

The aim of this study was to enhance the role of the two miRNAs in NMSC in correlation with the tumor development and evolution.

## 2. Materials and Methods

### 2.1. Samples Collection of NMSC Patients

In the Oral and Maxillofacial Surgery Department in Cluj-Napoca, Romania, 38 patients with highly-suspect head and neck NMSC lesions were examined. All of them were informed about the protocol of the study and consented to participate. The Ethical Committee of the Emergency County Hospital Cluj-Napoca, Romania (39109/A/0001/UK/R 306/08.01.2020), and the University of Medicine and Pharmacy Cluj-Napoca, Romania (562/2.12.2019), approved the study. Samples of malignant and adjacent tissue have been taken from each patient. After the initial check from a pathologist, samples were collected in liquid nitrogen tanks and frozen at −170 °C. Three patients were excluded after receiving pathological reports because their diagnose differed from NMSC.

### 2.2. RNA Isolation and Extraction

Total RNA was extracted and isolated from all 38 NMSC match paired (tumor and adjacent) tissue samples using the phenol-chloroform (Trireagent) method, according to the manufacturer’s protocol. The concentration of RNA was measured by the NanoDrop-1000 spectrophotometer and ranged to 50 ng/μL.

### 2.3. cDNA Synthesis and Quantitative Real Time RT-PCR

MiRNA expression levels were detected in *n* = 38 (*n* = 38 tumor and *n* = 38 adjacent) NMSC matched-pair tissue samples.

The cDNA synthesis was performed using a 7.5 μL of reverse transcription mixture containing 0.72 μL of RT primer, 25 ng of total RNA, and 0.5 μL of MultiScribe Reverse Transcriptase, 0.75 μL Reverse Transcription Buffer (10×), 0.075 μL dNTPs (100 mM), and 0.1 μL of RNase Inhibitor according to the TaqMan MicroRNA Reverse Transcription Kit (Applied Biosystems, Waltham, MA, USA) protocol. The cDNA mixture was incubated in PCR tubes at 16 °C for 30 min, 42 °C for 30 min, and 85 °C for 5 min.

qRT-PCR was performed in a total volume of 10 μL using 5 μL of cDNA (diluted 1:3 with nuclease-free water), 5.03 TaqMan Fast Advanced Master MIX (Applied Biosystems), and 0.47 μL primer for each miRNA in ViiA7 (Applied Biosystems) PCR machine. The primers of qRT-PCR were listed in Table 1. The reactions were set up as follows: the initial denaturation step at 50 °C for 2 min and 95 °C for 2 s, followed by 40 cycles of 95 °C for 1 s, and 60 °C for 20 s.

The expression level of each miRNA was calculated by threshold cycle (CT). The relative expression level was calculated using –ΔΔCT and fold change 2^−ΔΔCT. A median expression sample among all samples was chosen as a calibrator, and U6 and RNU48 as the controls for miRNAs expression (Table 1).

### 2.4. Statistical Analyses

Qualitative data was displayed as frequencies and percentages. Skewed continuous data was presented as medians, interquartile ranges, and boxplots with bee swarm plots. Normality of the data was verified with quantile-quantile plots and Shapiro—Wilk tests. Comparisons between groups concerning qualitative data were performed using the chi-squared and Fisher’s exact tests. Comparisons between two independent groups regarding skewed continuous data were performed with the Wilcoxon rank sum test. For all statistical tests, the 0.05 significance level was used and two-tailed *p* values. All analyses were conducted in an R environment for statistical computing and graphics (R Foundation for Statistical Computing, Vienna, Austria), version 4.1.2 [44].

## 3. Results

The cases were distributed according to the histopathological report, with 18 patients being diagnosed with BCC, 18 with SCC, and 2 with BSC. In more than 80% of cases, the tumor was classified as Clark IV or V, and more than 60% of SCC were T3/T4. As a negative predictive factor, perineural invasion was observed in almost half of the cases of SCC. The complete set of data can be analyzed in Table 2.

**Figure 1:**
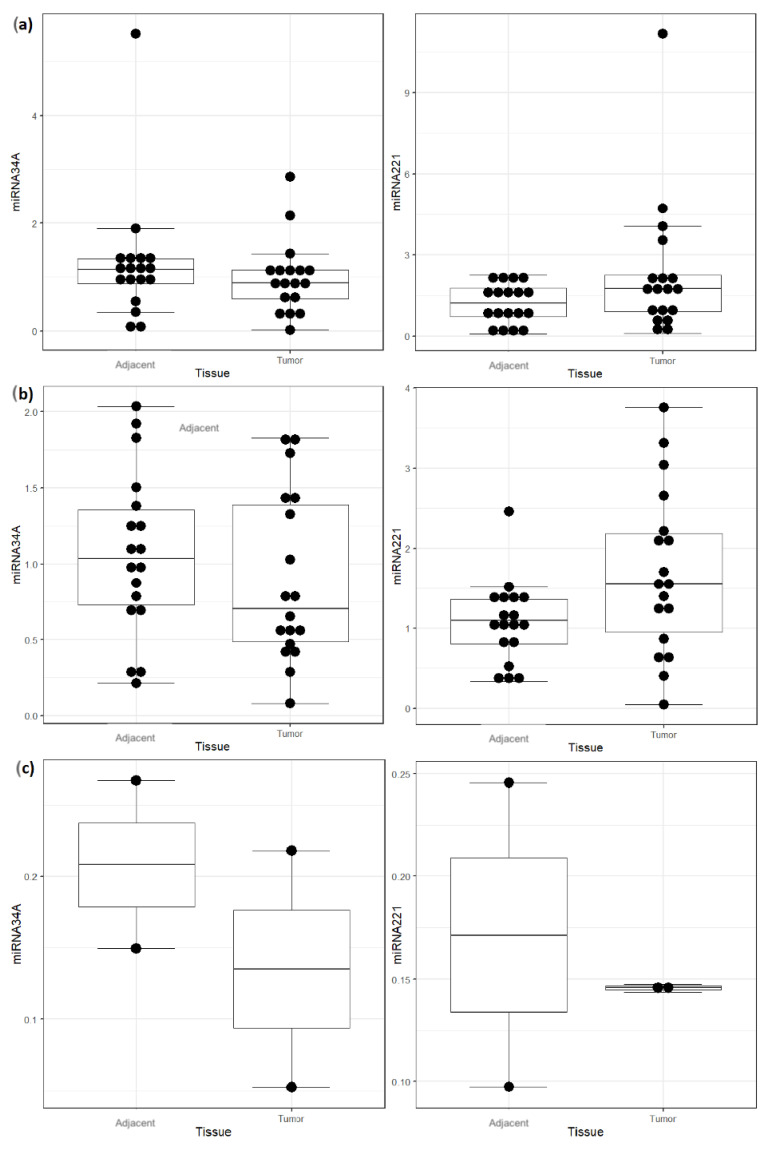
Comparison between tumoral and normal tissue regarding miRNA-34A and miRNA-221 expressions. (**a**) basocellular carcinoma, (**b**) squamous cell carcinoma, (**c**) baso-squamous cell carcinoma.

Compared to adjacent tissue, miRNA-34a shows an altered expression without statistical significance in all types of NMSC. The tumors were shown to express higher levels of miRNA-221 than adjacent tissue only in the SCC cases with statistical significance (*p* < 0.05) (Table 3) (Figure 1).

After histopathology evaluation, the status of the surgical margins was discovered to be positive in eight cases (R1). Compared to R0 patients, the levels of miRNA-221 were two times higher in these cases. The results are statistically significant (*p* < 0.05) (Table 4).

## 4. Discussion

MiRNA have been extensively studied in the field of skin malignancies, mostly in melanoma but also in other tumor types. Melanoma has represented an important study focus for miRNAs because of the tumoral heterogeneity which determines highly aggressive features and poor prognostic markers. One of the earliest predictive factors for miRNA-221 was discovered a few years ago, according to the miRNA expression in tumor tissue or body fluids. In addition, the expression of other miRNAs, including miRNA-NA-203, miRNA-10b, miRNA-200b, and miRNA-155, has been linked to metastasis and unfavorable results. MiRNA-675, miRNA-204, and the interaction between miRNA-135 and large tumor suppressor kinase 2 (LATS2) have all been proposed as potential therapeutic targets. Three miRNAs have been suggested as predictive biomarkers for a decreased survival rate: miRNA-125b, miRNA-200c, and miRNA-205. MiRNA-10b, miRNA-16, and miRNA-21 have been linked to a poor prognosis in larger studies which included over 25 miRNAs. The mechanism of therapeutic resistance is another function of miRNAs in melanoma patients and specific miRNAs, such as miRNA-92a-1-5p and miRNA-708-5p, have been correlated to particular genes [2].

Human tumor tissues, including those from melanoma, liver cancer, breast cancer, prostate cancer, colorectal cancer, and acute myeloid leukemia have been found to overexpress miRNA-221. Furthermore, distinct tumor-related factors, such as metastasis, tumor capsular infiltration, advanced tumor stage, and a poor prognosis are all associated with high levels of miRNA-221 expression. In the epithelial-to-mesenchymal transition (EMT), the role of miRNA-221 is crucial, due to its basal-like subtype-specific miRNA function which upregulates the expression of genes specific to mesenchymal cells while downregulating the expression of genes specific to epithelial cells. MiRNA-221 also promotes cell invasion and migration. The transcription of miRNA-221 can be directly induced by the basal-like transcription factor FOSL1. With the inhibition of mitogen-activated or extracellular signal-regulated protein kinase (MEK), the levels of miRNA-221 decrease. Targeting the 3′-UTR of the trichorhinophalangeal syndrome type 1(TRPS1) gene is necessary for the miRNA-221-mediated decrease in E-cadherin. By directly suppressing the expression of zinc finger E-box-binding homeobox 2 (ZEB2), TRPS1 prevents EMT. As a result, miRNA-221 may have a role in the aggressive clinical behavior of different forms of cancer [45]. MiRNA-221 can selectively target PTEN and reduce the production of PTEN protein, a well-known tumor suppressor in human malignancies. The PTEN gene, which is found on chromosome 10q23.31, has a role in the control of cellular proliferation, apoptosis, and metastasis [31,46,47,48,49].

Our research confirms the results of other authors and demonstrates the major involvement of miRNA-221 in the arising and evolution of NMSC. The role of miRNA221 as a predictive factor was discovered a few years ago, based on its expression evidenced in tumor tissues or body fluids, but only for SCC [50,51,52]. MiRNA221 expression in tumor samples was increased in contrast with the normal tissues, according to the measurements made on SCC clinical samples by Gong et al. [31]. MiRNA221 controls the migration, colony formation, and cell proliferation of SCC cells, which are essential for developing tumors. In our research, the levels of miRNA-221 were increased for both SCC and BCC but with statistical significance only increased for SCC. This can be explained by the small sample size of the patients. We think that further studies with larger size samples are necessary in order to demonstrate if this upregulation of miRNA-221 is specific only for SCC or for all NMSC. If it is upregulated only in SCC, it could be a key investigation in the differentiation of the SCC from the others NMSC.

Additionally, the miRNA-221 level was two-times increased in cases where positive margins were identified after the surgical resection. From our research, we are the first to highlight this particularity and the potential contribution of miRNA-221 in the microscopical local invasion. Because the tumor involvement of the surgical margins is one of the most significant negative predictive factors, the detection of miRNA-221 in tumor samples should guide the clinician to extend the macroscopical limits of the surgery in order to avoid further complications, such as local recurrence. The ideal scenario will be to detect this miRNA in the patient serum and to guide the surgical intervention in accordance with its level. Additionally, this could be helpful in the diagnosis process, if miRNA-221 is specific only for SCC and for the prognosis of the disease.

More important research regarding miRNA-221 were done for malignant melanoma, which is the other category of skin malignancy. According to Kanemaru et al., melanoma patients had considerably higher serum levels of miRNA-221 and these levels could lead to the identification of the disease. However, they did not focus on the connection between the expression level of miRNA-221 in serum and the prognosis of malignant melanoma. Li P. et al. discovered that tissue miRNA-221 expression was significantly higher in those patients with cutaneous malignant melanoma and that high levels of miRNA-221 expression were particularly linked to poor differentiation, tumor thickness, T classification, N classification, metastasis, and advanced clinical stage. These results suggest that miRNA-221 overexpression may be able to play a major role in the rise of cutaneous malignant melanoma. Additionally, they stated that patients with high levels of miRNA-221 expression had decreased 5-year survival and disease-free survival rates than patients with lower levels of the gene, suggesting the potential role of miRNA-221 as a molecular marker to predict a patient’s prognosis for cutaneous melanoma [45,50]. We think that 5-year survival and disease-free survival rates should also be studied for NMSC in accordance with the miRNA-221 level.

No other negative predictive factors, such as the presence of the lymph node or distant metastasis, the perineural invasion, lymphatic or vascular invasion, or advanced-stage tumors, were associated with miRNA-221. In our research, no patient presented regional or distant metastasis, therefore, we could not evaluate the role of miRNA-221 in the development of metastasis. In case regional metastasis is present, the survival rate is reduced dramatically by half. Because prophylactic neck dissection with superficial parotidectomy and sentinel node biopsy are not routinely done in NMSC, a biological marker to predict the development of metastasis will be ideal.

Our study has also found altered levels of miRNA-34a in BCC, SCC, and basosquamous cell carcinoma tumor samples, as compared with the adjacent ones, which confirms the role of miRNA-34a in oncogenesis and the development of non-melanoma skin malignancy. The role of miRNA-34a in skin cancer development has been highlighted in a few studies. MiRNA-34a was found in serum samples from BCC patients by Hu P et al. They discovered that patients with BCC had lower levels of miRNA-34a expression in comparison with the general population and they enhanced a connection between miRNA expression and tumor cell diameter, lymph node metastasis, and different histological subtypes of BCC. Distinct parameters, such as the median progression-free survival, overall survival time, and survival rate, were lower in the downregulated group. Overall, patients with basal cell carcinoma exhibiting a decreased miRNA-34a expression had a poor prognosis [43]. We think that larger studies are necessary to confirm the previous results. Additionally, the role of miRNA-34a should be extended for SCC and BSC because we found altered levels of miRNA in both of them. MiRNA-34a expression was discovered to be downregulated in SCC both in vivo and in vitro, according to Lefort et al. [53]. The rate of metastasis for BCC is very low so we think that having a biological marker for this is not so important. From other points of view, the behavior of the tumor is different for the nodular type and the invasive or Morpheus one. Future research should also concentrate on the expression of miRNA-34a in different types of BCC.

The development and evolution of several other malignant tumors, including breast and esophageal cancer and lung adenocarcinoma, are strongly correlated with the downregulation of miRNA-34a, which is a predictive risk factor. Regarding the mechanism of action, research has demonstrated that the irreversible inactivation of miRNA-34a may be another defensive mechanism of tumor cells [2,3]. In UV-exposed keratinocytes, TP53 gene mutations are frequent and have a role in apoptotic resistance in skin cancer [54,55,56]. By influencing the TP53 gene action, miRNA-34a has effects on distinct cell processes, such as the growth arrest, senescence, and apoptosis [57,58]. A method of influencing cell death signaling resistance in non-melanoma cancers may be represented by the TP53 gain-of-function mutations targeting. Studies have also shown that miRNA-34a can cooperate with other tumor suppressors to influence the cell cycle and promote cell arrest in the G1/S phase, subsequently suppressing the abnormal expansion of tumor cells, as in non-small cell lung cancer [53]. Nowadays, the diagnosis of skin malignancy is established by clinical examination, dermoscopy, imaging, and finally by biopsy. The detection of miRNA-34a in blood samples could play an important role in NMSC diagnosis through non-invasive techniques [59,60,61].

The flaw of our research is represented by the small size of the samples, due to the necessity of dividing the probes into tumor samples for BCC, BSC, and SCC and adjacent tissue. Further studies are necessary. Future research should focus on altering miRNA-221 and miRNA-34a levels in the serum. This may represent a very important part in determining a tumor’s future behavior and preventing an incomplete surgical excision. For example, serum samples from melanoma patients were used to detect and measure the levels of circulating miRNA-221. When compared to the samples from patients with melanoma in situ, patients with invasive melanoma had considerably higher levels of miRNA-221 and these results were connected with the tumor thickness. Furthermore, longitudinal research discovered that the levels of miRNA-221 have a tendency to decrease after the surgical removal of the initial tumor and to increase once more during recurrence [45].

## 5. Conclusions

Non-melanoma skin cancers represent a potential challenge because of their arising incidence and rapidly developing growth. Unveiling the molecular mechanisms leads to a better understanding of the malignant landmark, such as tumorigenesis, the process of metastasis, and evolution, while also contributing to the improvement of novel therapeutic keys. The miRNAs, which are represented by the small non-coding RNAs influencing gene expression at the post-transcriptional level, have gained a particular interest nowadays. MiRNA-221 and MiRNA-34a can be easily detected from tumor samples in all types of NMSC, having a role in the oncogenesis and evolution of the disease, and could play a major part in the treatment decision.

This study demonstrated that the overexpression of miRNA-221 is present in SCC tissue and is associated with higher rates of positive margins after surgical resection and requires particular interest. The detection of miRNA-34a in blood samples could enhance a significant contribution in NMSC diagnosis through non-invasive techniques. Further studies to investigate the potential role of miRNA 221 in differential diagnosis between SCC and BCC and the role in the development of metastasis are necessary. Additionally, the role of miRNA-34a in the differentiation of the BCC subtypes should be further investigated.

## Figures and Tables

**Table 1:** MiRNA Primers used in the study.

Assay Name	Assay ID
hsa-miRNA-34-5p	000426
hsa-miRNA-221-3p	000524
U6 snRNA	001973
RNU48	001006

**Table 2:** Nonmelanoma skin cancer participants’ characteristics.

Histopathology:	BCC (*n* = 18)	BSC (*n* = 2)	SCC (*n* = 18)	*p*-Value
Age, median (IQR)	70.5 (62–78)	76 (74.5–77.5)	76.5 (65.25–84.5)	0.454
Sex (F), *n* (%)	10 (55.56)	1 (50)	7 (38.89)	0.747
Breslow, median (IQR)	5.45 (3.67–9.38)	4.1 (3.55–4.65)	7 (5–12)	0.439 {0.659/0.747/0.156}
Clark, *n* (%)				0.267
II:	1 (5.56)	0 (0)	1 (5.88)	
III:	1 (5.56)	0 (0)	0 (0)	
IV:	7 (38.89)	1 (50)	2 (11.76)	
V:	9 (50)	1 (50)	14 (82.35)	
Clark IV/V, *n* (%)	16 (88.89)	2 (100)	16 (88.89)	
T3/4, *n* (%)	4 (22.22)	0 (0)	12 (66.67)	0.011
V1, *n* (%)	1 (5.56)	1 (50)	2 (11.11)	0.285
L1, *n* (%)	1 (5.56)	0 (0)	5 (27.78)	0.239
R1, *n* (%)	4 (22.22)	1 (50)	3 (16.67)	0.523
Pn1, *n* (%)	4 (22.22)	0 (0)	8 (44.44)	0.292

T, tumor stage; V, vascular invasion; L, lymphatic invasion, R, resection margin; Pn, perineural invasion; IQR, interquartile range.

**Table 3:** Comparison between tumoral and adjacent tissue regarding miRNA-34a and miRNA-221 expression.

Tumor Type and miRNA	Normal (*n* = 18)	Tumoral (*n* = 18)	Difference (95% CI)	*p*-Value
Basocellular carcinoma (BCC)				
miRNA-34A, median (IQR)	1.14 (0.88–1.34)	0.89 (0.6–1.13)	0.24 (−0.2–0.53)	0.279
miRNA-221, median (IQR)	1.21 (0.72–1.77)	1.75 (0.91–2.25)	0.54 (−1.37–0.2)	0.161
Squamous carcinoma (SCC)				
miRNA-34A, median (IQR)	1.03 (0.73–1.36)	0.7 (0.49–1.39)	0.33 (−0.25–0.57)	0.308
miRNA-221, median (IQR)	1.09 (0.8–1.36)	1.55 (0.95–2.19)	0.46 (−1.12–−0.03)	0.04
Basosquamous carcinoma (BSC)				
miRNA-34A, median (IQR)	0.21 (0.18–0.24)	0.14 (0.09–0.18)	0.07 (−0.07–0.21)	1
miRNA-221, median (IQR)	0.17 (0.13–0.21)	0.15 (0.14–0.15)	0.03 (−0.05–0.1)	1

miRNA, micro ribonucleic acid; IQR, interquartile range.

**Table 4:** Synthesis: miRNA-34A tumor tissue, miRNA-221 tumor tissue, depending on R.

R1:	R1 (*n* = 8)	R0 (*n* = 30)	Difference (95% CI)	*p*
miRNA-34A tumor tissue, median (IQR)	1.05 (0.8–1.59)	0.66 (0.42–1.13)	0.39 (−0.08–0.98)	0.14 [n1 = 8, n2 = 30]
miRNA-221 tumor tissue, median (IQR)	3.64 (1.78–4.22)	1.46 (0.67–1.95)	2.18 (0.36–3.16)	0.019 [n1 = 8, n2 = 30]

R, resection margin; IQR, interquartile range.

## Data Availability

The data presented in this study are available on request from the corresponding author.
